# 193. Standard of Care versus Alternative Beta-lactams for Treatment of Infections due to *Serratia marcescens*

**DOI:** 10.1093/ofid/ofad500.266

**Published:** 2023-11-27

**Authors:** Sarah B Green, Joseph Torrisi, Shreena P Advani, Benjamin Albrecht, Jesse T Jacob, Kayla Ashley Jones, Sheetal Kandiah, Kristen Paciullo, Darshan Patel, Manish Patel, Sarah W Satola, Sujit Suchindran, Roland Tam, Ronald Trible, Trinh P Vu, Lucy S Witt, Jessica Howard-Anderson, Ahmed Babiker

**Affiliations:** Emory University Hospital, Atlanta, Georgia; Grady Health System, Atlanta, Georgia; Grady Health System, Atlanta, Georgia; Emory University Hospital, Atlanta, Georgia; Emory University School of Medicine, Atlanta, GA; Emory Healthcare, Atlanta, Georgia; Emory university school of medicine, Atlanta, Georgia; Emory Saint Joseph's Hospital, Atlanta, Georgia; Emory Johns Creek Hospital, Acworth, Georgia; Grady Health System, Atlanta, Georgia; Emory University School of Medicine, Division of Infectious Diseases, Atlanta, Georgia; Emory University School of Medicine, Atlanta, GA; Emory Healthcare, Atlanta, Georgia; Emory Saint Joseph's Hospital, Atlanta, Georgia; Emory University Hospital Midtown, Atlanta, Georgia; Emory University, Atlanta, Georgia; Emory University, Atlanta, Georgia; Emory University School of Medicine, Atlanta, GA

## Abstract

**Background:**

The threat of inducible resistance and treatment failure positioned cefepime and carbapenems as the standard of care (SOC) treatment agents for infections due to AmpC-producing Enterobacterales regardless of antimicrobial susceptibility testing results. Recently published Infectious Diseases Society of America Guidance (IDSA) classifies the overall risk of clinically relevant AmpC expression for *Serratia marcescens* as low (< 5%). We aimed to compare the clinical outcomes of patients treated with a SOC antibiotic (carbapenem or cefepime) to those treated with an alternative beta-lactam agent for *S. marcescens* bloodstream infection (BSI).

**Methods:**

This multisite, retrospective study included patients from five hospitals in the Atlanta-Metropolitan area with *S. marcescens* BSIs between January 1, 2018 and December 31, 2022. Patients were included if they received at least 72 hours of therapy with a beta-lactam antibiotic whose in vitro susceptibility was confirmed. Patients with polymicrobial bacteremia, those receiving combination therapy for > 72 hours, or those with a diagnosis of endovascular infection were excluded. We used a desirability of outcome ranking (DOOR) analysis to determine the probability of having a more desirable outcome receiving SOC antibiotics compared to receiving an alternative beta-lactam agent. The DOOR analysis includes counting undesirable events (Figure 1) and was performed using an online calculator (https://methods.bsc.gwu.edu).

**Figure 1:**
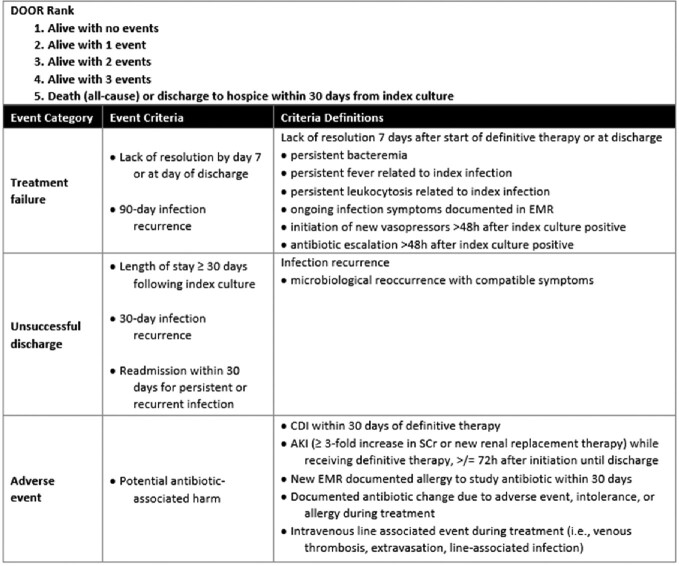
Primary endpoint - Desirability of outcome ranking. Abbreviations: AKI = acute kidney injury; CDI = Clostridioides difficile infection; EMR = electronic medical record; SCr = serum creatinine

**Results:**

Of the 175 *S. marcescens* blood cultures reviewed, 66 patients met criteria for study inclusion, 43 in the SOC group and 23 in the alternative group. Baseline characteristics were comparable in both groups (Figure 2). The DOOR distribution between treatment groups was similar (Figure 3) and there was no significant difference in the probability of a more desirable outcome for patients who received an alternative beta-lactam compared to SOC (54.2% [95% CI, 40.3 - 67.5%]; Figure 4).

**Figure 2:**
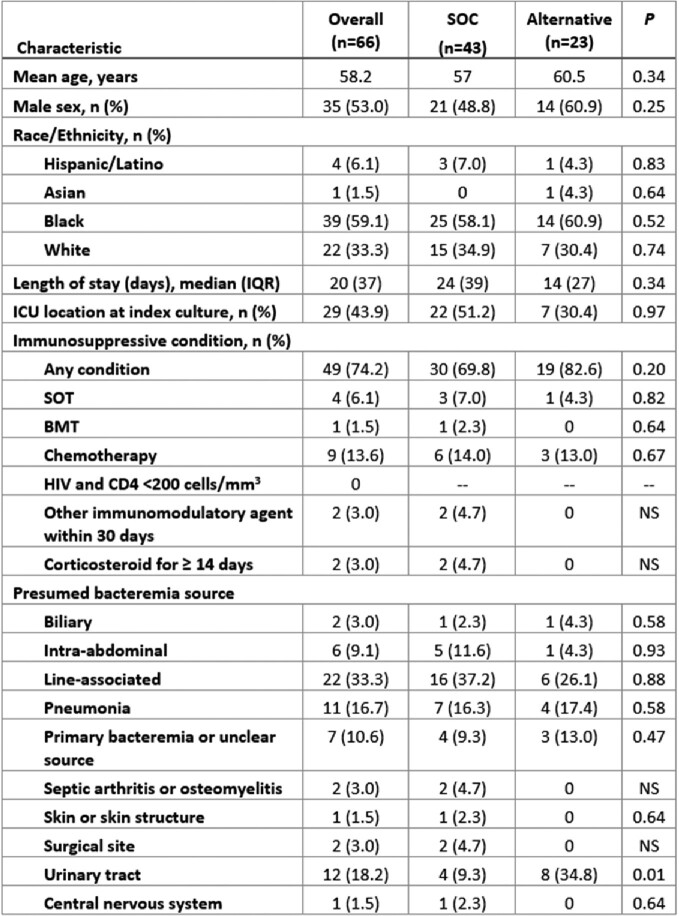
Baseline patient characteristics. Abbreviations: BMT = bone marrow transplant within previous 12 months; Corticosteroid = ≥15 mg prednisolone daily equivalents; HIV = human immunodeficiency virus; ICU = intensive care unit; IQR = interquartile range; NS = non-significant; SOC = standard of care; SOT = any history of solid organ transplant

**Figure 3:**
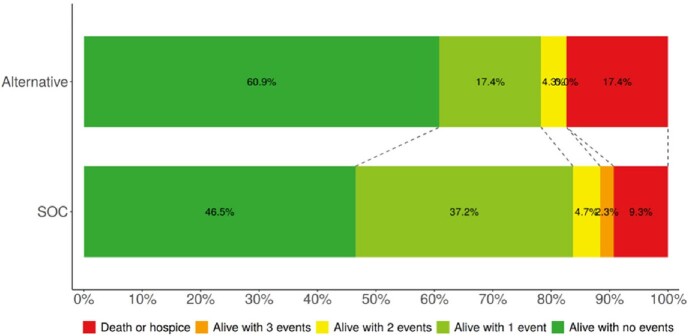
Desirability of Outcome Ranking Distribution Summary

Desirability of outcome ranking distribution for the standard of care and alternative beta-lactam treatment groups; SOC = standard of care

**Figure 4:**
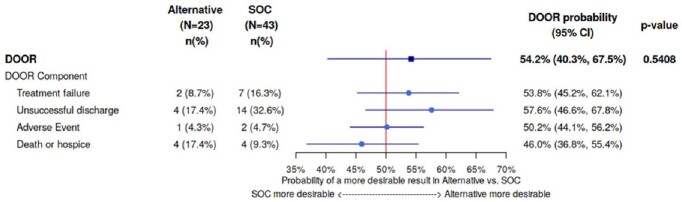
Desirability of Outcome Ranking Probability Summary

Forest plot of desirability of outcome ranking probabilities by overall treatment group and individual ranking components; CI = confidence interval, DOOR = desirability of outcome ranking, SOC = standard of care

**Conclusion:**

The overall outcome of patients receiving alternative beta-lactam antibiotics was similar to those receiving SOC antibiotics in this study. These results support use of alternative beta-lactams for treatment of susceptible *S. marcescens* BSIs as recommended by IDSA guidance.

**Disclosures:**

**Joseph Torrisi, PharmD, BCIDP**, Clinical Care Operations: Honoraria **Ahmed Babiker, MBBS**, Roche: Advisor/Consultant

